# Performance of the Southend pre-test probability score (PTPS) for giant cell arteritis in a fast-track clinic in Western Australia

**DOI:** 10.1093/rap/rkac055

**Published:** 2022-07-26

**Authors:** Mmoloki Mathake, Julia Murdoch, Jean-Louis DeSousa, Andrew Taylor, Helen Keen

**Affiliations:** Rheumatology, Fiona Stanley Hospital, Murdoch; Rheumatology; Ophthalmology, Royal Perth Hospital, Perth, WA, Australia; Rheumatology; Rheumatology, Fiona Stanley Hospital, Murdoch

Key Message

- The Southend pre-test probability score might be useful in risk stratifying patients presenting to GCA fast-track clinics.

Dear Editor, We report the performance of the Southend pre-test probability score (PTPS) for GCA in our GCA assessment clinic. GCA is the most common large vessel vasculitis. Intense vascular inflammation leads to myointimal proliferation and potential vessel occlusion, which can result in major ischaemic events, such as blindness and stroke. Therefore, GCA is considered a medical emergency, necessitating timely and accurate diagnosis and intervention [1, 2]. GCA most frequently presents with headache, which is a common and non-specific presentation. Associated features can include jaw claudication, scalp tenderness, visual disturbances and non-specific constitutional symptoms; however, the symptoms at presentation are heterogeneous. Physical signs, when present, can include thickened or tender temporal arteries on palpation in addition to diminished or absent pulses [1]. Inflammatory markers are generally elevated, but normal inflammatory markers do not preclude the diagnosis. For these reasons, diagnosis can be challenging.

Objective confirmation of the diagnosis is pursued to justify the use of aggressive and long-term immunosuppression. Historically, TA biopsy has been considered the gold standard for diagnosis for GCA. TA biopsy has a high specificity but low sensitivity (as low as 39%), is invasive and can be difficult to obtain in a timely manner [1]. Recently, imaging modalities such as US, PET scan, MRI and CT scan have been endorsed as diagnostic tools in international guidelines, but they assume local competency and ease of access [1–3]. Furthermore, the pre-test probablity of GCA is essential to consider when assessing the clinical utility of these imaging modalities, because none provides stand-alone definitive confirmation of the disease.

The pre-test probability of disease is a crucial component of GCA assessment and clinical decision-making. Therefore, a clinical tool to define the probability of GCA objectively is likely to be very useful. Recently, the Southend pre-test probability score (PTPS) has been developed to aid GCA assessment [4, 5]. The tool was developed retrospectively and has not been tested in different populations. In this study, we aimed to apply the PTPS to patients seen through our GCA fast-track clinic in Western Australia to assess the utility of this score in risk stratifying patients with suspected GCA.

Patients presenting to the Royal Perth Hospital GCA fast-track clinic were consented for prospective data collection. GCA was defined as clinical diagnosis of GCA at 6 months, based on the opinion of clinicians, formulated on history, examination, TA US and TA biopsy or additional imaging in select cases. We calculated the Southend PTPS retrospectively from the data collected prospectively over the 18 months between November 2019 and May 2021. Using the PTPS, we risk stratified patients into low-risk (PTPS < 9), intermediate-risk (PTPS = 9–12), and high-risk (PTPS > 12) groups and correlated these with the final clinical diagnosis. We then dichotomized the PTPS into low-risk or intermediate/high-risk groups to determine the sensitivity, specificity, positive predictive value and negative predictive value.

Data were analysed from 104 patients over the specified time frame. The demographics and the prevalence of features of the Southend PTPS are presented in Table 1. The clinical diagnosis of GCA at 6 months occurred in 25, giving a prevalence of 24%. Using the PTPS, 45 (43%) of the 104 patients were classified as low risk, 34 (32%) intermediate risk and 25 (24%) high risk for GCA. In the low-risk category, GCA prevalence was 0%, in the intermediate-risk group 15% and in the high-risk category 80% (*P* < 0.001).

**Table 1:** Baseline demographics and features of patients with GCA compared with the total number of patients

Demographics	Number of patients (*n* = 104)	Patients with GCA (total *n* = 25 of 104)
Age, years		
<49	4	0
50–60	20	4
61–65	19	3
>66	61	18
Gender		
Male	30	12
Female	74	13
Duration of symptoms, weeks		
>24	13	2
12–24	9	1
6–12	16	2
<6	66	20
CRP, mg/l^a^		
0–5	25	4
6–10	14	0
11–25	26	4
>25	36	15
Symptoms		
Headache		
No	11	4
Yes	93	21
Polymylagia		
No	87	19
Yes	17	6
Constitutional		
No	77	19
Single	22	6
Combination	5	0
Ischaemic		
No	86	13
Yes	18	12
Signs		
Visual		
No	92	17
Yes	12	8
TA abnormality		
No	85	16
Tenderness	10	1
Thickening	4	3
Pulse loss	5	5
Extracranial artery abnormality		
No	102	23
Thickening	2	2
Bruit	0	0
Pulse loss	0	0
Cranial nerve palsy		
No	104	25
Yes	0	0
Alternative		
Infection	12	0
Cancer	5	2
Systemic rheumatic diseases	1	0
Head and neck pathology	0	0
Other	10	2

a The CRP values for three patients in the control group and four in the GCA group were missing from the records (this would not have changed the category of the patients).

The Southend PTPS (dichotomized) had a sensitivity of 100%, specificity of 56.9%, positive predictive value of 42.3% and negative predictive value of 100%.

In conclusion, the PTPS (applied retrospectively) successfully stratified patients referred to our fast-track clinic into high and low risk for GCA, when using the clinical diagnosis as the gold standard. Our study provides external validation to the currently published work. This tool might be useful in risk stratifying patients who are undergoing specialist assessment for GCA in the fast-track clinic setting; the negative predictive value suggests that this tool is valuable to exclude GCA, but this needs to be tested prospectively.

*Funding:* No specific funding was received from any bodies in the public, commercial or not-for-profit sectors to carry out the work described in this article.

*Disclosure statement:* The authors declare no conflict of interest.

## Data Availability

The datasets generated during and/or analysed during the current study are available from the corresponding author on reasonable request.
